# Comparing the Perspectives of Generative AI, Mental Health Experts, and the General Public on Schizophrenia Recovery: Case Vignette Study

**DOI:** 10.2196/53043

**Published:** 2024-03-18

**Authors:** Zohar Elyoseph, Inbar Levkovich

**Affiliations:** 1 Department of Brain Sciences, Faculty of Medicine, Imperial College London, London, United Kingdom; 2 The Center for Psychobiological Research, Department of Psychology and Educational Counseling, Max Stern Yezreel Valley College, Emek Yezreel, Israel; 3 Faculty of Graduate Studies, Oranim Academic College, Kiryat Tiv'on, Israel

**Keywords:** schizophrenia, mental, prognostic, prognostics, prognosis, ChatGPT, artificial intelligence, recovery, vignette, vignettes, outcome, outcomes, large language models, language model, language models, LLM, LLMs, NLP, natural language processing, GPT, Generative Pre-trained Transformers

## Abstract

**Background:**

The current paradigm in mental health care focuses on clinical recovery and symptom remission. This model’s efficacy is influenced by therapist trust in patient recovery potential and the depth of the therapeutic relationship. Schizophrenia is a chronic illness with severe symptoms where the possibility of recovery is a matter of debate. As artificial intelligence (AI) becomes integrated into the health care field, it is important to examine its ability to assess recovery potential in major psychiatric disorders such as schizophrenia.

**Objective:**

This study aimed to evaluate the ability of large language models (LLMs) in comparison to mental health professionals to assess the prognosis of schizophrenia with and without professional treatment and the long-term positive and negative outcomes.

**Methods:**

Vignettes were inputted into LLMs interfaces and assessed 10 times by 4 AI platforms: ChatGPT-3.5, ChatGPT-4, Google Bard, and Claude. A total of 80 evaluations were collected and benchmarked against existing norms to analyze what mental health professionals (general practitioners, psychiatrists, clinical psychologists, and mental health nurses) and the general public think about schizophrenia prognosis with and without professional treatment and the positive and negative long-term outcomes of schizophrenia interventions.

**Results:**

For the prognosis of schizophrenia with professional treatment, ChatGPT-3.5 was notably pessimistic, whereas ChatGPT-4, Claude, and Bard aligned with professional views but differed from the general public. All LLMs believed untreated schizophrenia would remain static or worsen without professional treatment. For long-term outcomes, ChatGPT-4 and Claude predicted more negative outcomes than Bard and ChatGPT-3.5. For positive outcomes, ChatGPT-3.5 and Claude were more pessimistic than Bard and ChatGPT-4.

**Conclusions:**

The finding that 3 out of the 4 LLMs aligned closely with the predictions of mental health professionals when considering the “with treatment” condition is a demonstration of the potential of this technology in providing professional clinical prognosis. The pessimistic assessment of ChatGPT-3.5 is a disturbing finding since it may reduce the motivation of patients to start or persist with treatment for schizophrenia. Overall, although LLMs hold promise in augmenting health care, their application necessitates rigorous validation and a harmonious blend with human expertise.

## Introduction

### Background

Schizophrenia is a major contributor to mental health–related disability worldwide and exerts a profound effect on patients and society [1]. It has a major impact on life expectancy and quality of life, and its repercussions extend to family and caregivers [2]. The disorder presents a complex array of symptoms, both “positive” (eg, delusions and hallucinations) and “negative” (eg, emotional flatness and social withdrawal) [3]. Negative symptoms are especially resistant to current treatments [4]. Despite the complexity and impact of this disorder, a subset of individuals with schizophrenia may have a favorable prognosis; exhibit symptom reduction; and achieve positive outcomes in education, employment, and relationships [3].

A systematic review based on 37 studies that examined outcomes in first-episode psychosis [5] determined that 42% of patients experienced favorable outcomes. Similarly, an analysis of 114 follow-up studies to assess recovery rates in schizophrenia [6] yielded recovery rates ranging from 11% to 33% for complete recovery and from 22% to 53% for social recovery. Another meta-analysis [7] encompassing 50 pertinent studies revealed that approximately one-seventh of individuals diagnosed with schizophrenia met our predefined criteria for recovery.

The beliefs held by therapists regarding their patients’ capacity for recuperation represent a complex and multifaceted conundrum [8]. From a pragmatic standpoint, a medical practitioner’s proficiency in ascertaining a patient’s prospective therapeutic trajectory (known as “prognosis” in the medical field) is a major clinical aptitude [9]. From an ethical perspective, clinicians are duty bound to elucidate potential perils and advantages to patients, thus facilitating a process of informed consent and collaborative decision-making [10]. Providing a nuanced yet candid prognosis enhances patient motivation and optimism when the likelihood of complete remission is high, while concurrently calibrating expectations in less promising scenarios [11-13]. Nevertheless, inherent values and presuppositions inevitably shape prognostic assessments [14,15]. The etiology and treatability of psychiatric disorders are framed by 2 opposing philosophical paradigms. Deterministic models, which view mental disorders as fixed biological anomalies, often adopt a pessimistic perspective on full recovery. In contrast, the recovery model approach is rooted in the belief that complete recovery is achievable. This perspective emphasizes personal empowerment, resilience, and community integration, focusing on an individual’s potential rather than solely on their symptoms [6,14,15]. Dogmatic adherence to either of these viewpoints carries the risk of engendering self-realizing outcomes. Hence, therapists must balance their understanding of empirical medical data by acknowledging the vast spectrum of human potentialities [16,17]. In November 2022, the generative artificial intelligence (AI) large language model (LLM) ChatGPT-3 was launched for free public use. Subsequently, in 2023, other LLMs such as Google Bard, Claude, and ChatGPT-4 were released. Although all these LLMs have been trained on vast data sets and have undergone alignment processes, as well as learning from user feedback, their differences stem from their unique learning algorithms; the nature of their training data; and the distinct approaches to alignment, user interaction, and learning from user feedback. These LLMs have permeated various facets of society, including political science, economics, health care, and biology [18,19]. Previous studies have examined the potential of LLMs in the context of applied psychology, focusing on basic clinical abilities [20-22] or on decision-making in complex clinical situations such as depression and suicide [23-25]. To the best of our knowledge, no study to date has assessed the extent to which generative AI can facilitate cure or recovery from mental health conditions. In contrast, extensive literature highlights the immense therapeutic value of therapists’ belief in their patients’ ability to recover [11-13], as well as the negative effects that ensue when a therapist does not believe that the patient’s condition can improve [26].

Recovery for individuals with prolonged mental health challenges is a multifaceted process subject to varied interpretations. From a clinical perspective, recovery emphasizes symptom reduction and impairment rectification [26,27]. In contrast, from lived experience, recovery represents an individualized, potentially ongoing trajectory toward reclaiming purpose, meaning, and active contribution, regardless of symptoms [27].

Years of rigorous theoretical and clinical research have revealed several mechanisms that assist patient recovery. One salient finding is the positive correlation between a strong therapeutic alliance and enhanced outcomes [28]. A meta-analysis of over 30,000 participants showed the therapeutic alliance was highly correlated with outcomes, regardless of therapy type [29]. The efficacy of psychotherapy is well documented [30-32]. Therapists’ belief in treatment potential significantly impacts outcomes [33]. Over the past decade, literature has consistently emphasized recovery-oriented practices for improving patient outcomes, including enhanced functioning, goal setting, reduced legal issues, and decreased hospital admissions [34,35]. Consequently, mental health services increasingly integrate recovery paradigms into treatment strategies [36]. However, an abrupt transition from a biomedical model to recovery orientation can challenge providers, often leading to continued paternalistic decision-making [37].

With the increasing integration of AI in health care, especially given its emerging capabilities in emotion detection and mental health risk assessment [20-25], it becomes imperative to scrutinize how different LLMs interpret human recovery potential. Such an inquiry gains heightened relevance in that both patients and professionals are increasingly relying on LLMs for consultations. Not only do such insights have the potential to shape the trajectory of patient care, but they can also play a pivotal role in psychoeducational endeavors, direction, and interventions.

This research is based on an examination of the perspectives of mental health professionals in Australia [38]. The study included 342 nurses, 564 psychiatrists, 424 general practitioners (GPs), and 228 clinical psychologists. It also incorporated the insights of 982 members of the general public. Respondents were presented vignettes depicting an individual diagnosed with schizophrenia and asked to indicate their perceptions regarding prognosis, long-term outcomes, and potential discrimination.

### Research Objectives

The research objectives were as follows:

1. To examine how different LLMs (ChatGPT-3.5, ChatGPT-4, Claude, and Bard) evaluate the prognosis of an individual with schizophrenia compared to the evaluations of mental health professionals (mental health nurses, clinical psychologists, psychiatrists, and GPs) and the general public.
2. To examine how different LLMs (ChatGPT-3.5, ChatGPT-4, Claude, and Bard) evaluate the positive and negative long-term outcomes of an individual with schizophrenia compared to the evaluations of mental health professionals (mental health nurses, clinical psychologists, psychiatrist, and GPs) and the general public.
3. To compare evaluations of the prognosis and positive and negative outcomes of an individual with schizophrenia between different types of LLMs (ChatGPT-3.5, ChatGPT-4, Claude, and Bard).

## Methods

### AI Procedure and Data Collection

During the month of August 2023, we examined the following LLMs:

- *Bard* (Google; subsequently rebranded as Gemini) [39] uses the LaMDA language model, trained on the expansive Infiniset data set amalgamating over 1.5 trillion words from diverse web-based sources including C4-derived content, Wikipedia, programming documentation, and public forum dialogue. LaMDA was initially pretrained on extensive public and web text corpora, leveraging a transformer-based neural architecture and unsupervised learning to process language and formulate pertinent responses.
- *Claude* (Anthropic) [40] targets a beneficial, inoffensive, and truthful output using a constitutional approach. Its 12+ billion–parameter transformer model aims to ethically tackle linguistic complexity. Its training emphasized educational data curation and the alignment of model actions with human values and safety considerations, potentially enhancing reliability. A paid Claude subscription recently launched at US $20 per month. Our study used the free version.
- *ChatGPT-3.5* (OpenAI) [41] exemplifies a 175 billion–parameter transformer model demonstrating human-like language aptitude given its far-reaching internet-derived training data. It was designed to expand comprehension and text generation capabilities using immense data sets for advanced language understanding and tailored response building functionalities.
- *ChatGPT-4* (OpenAI) [42] aims to improve upon previous versions in areas such as safety, accuracy, and content policy adherence. Relative to ChatGPT-3.5, OpenAI claims an over 80% decrease in rule-violating responses and a 40% improvement in factual correctness for this latest model. As part of the training process, OpenAI solicited abundant feedback from early users and collaborated with over 50 AI ethics experts. However, full details on the architecture changes and training data incorporated have not been disclosed publicly. Without access to such technical specifics, independent auditing remains limited regarding the capabilities of ChatGPT-4. This model costs US $20 per month.

The case vignettes and a list of follow-up questions were introduced for each of the LLMs. To address the generative nature of the models, which produces different responses to similar questions, each vignette was passed through each model 10 times. We compared the LLMs’ data for schizophrenia prognosis to the norms used by mental health professionals and to the views of the general public, as reported by Caldwell and Jorm [38].

### Input Source

In this investigation, we adopted the text vignette methodology used by Jorm et al [43] and Caldwell and Jorm [38] to examine the beliefs of mental health professionals (mental health nurses, clinical psychologists, psychiatrists, and GPs) and the general public regarding the efficacy of interventions for schizophrenia. In the study, 2 vignettes were entered into the LLMs’ interface. The 2 vignettes differed only in the gender of the main character. Each vignette was evaluated 10 times in ChatGPT-3.5, ChatGPT-4, Bard, and Claude, for a total of 80 evaluations, each in a new tab.

### Vignette

The full text of the vignettes is as follows:

*Mary/Jone is 24 and lives at home with her/his parents. She/he has a few temporary jobs since finishing school but is now unemployed. Over the last six months she/he has stopped seeing her/his friends and has begun locking herself/himself in her/his bedroom and refusing to eat with the family or to have a bath. Her/His parents also hear her/him walking about her bedroom at night while they are in bed. Even though they know she/he is alone, they have heard her/him shouting and arguing as if someone else is there. When they try to encourage her/him to do more things, she/he whispers that she/he won’t leave home because she/he is being spied upon by the neighbor. They realize she/he is not taking drugs because she/he never sees anyone or goes anywhere.*

### Measures

After entering each of the 2 vignettes to the LLMs, we submitted 6 questions taken from Jorm et al [43]:

*Q1: Describe what, if anything, is wrong with Mary/Jone?*

*Q2: What will be helpful for Mary/Jone?*

*Q3: What is the most likely result for Mary/Jone given that she/he received the sort of professional help you believe is most appropriate? A. Full recovery with no further problems; B. Full recovery, but problems will probably recur; C. Partial recovery; D. Partial recovery, but problems will probably recur; E. No improvement; F. Get worse*.

*Q4: What is the most likely result for Mary/Jone if she/he does not receive any professional help? A. Full recovery with no further problems; B. Full recovery, but problems will probably recur; C. Partial recovery; D. Partial recovery, but problems will probably recur; E. No improvement; F. Get worse*.

*Q5: How do you think Mary/Jone will be in the long term compared to other people in the community? Rate each of the following statements according to this scale: more likely, just as likely, or less likely*. Negative long-term outcomes*: A. will be violent; B. will drink too much; C. will take illegal drugs; D. will have unsatisfactory friendships; E. will attempt suicide*. Positive long term outcomes: *F . will be understanding of other people’s feelings; G. will have a good marriage; H. will be a caring parent; I. will be a productive worker. J. will be creative or artistic*.

*Q6. Do you think Mary/Jone will be discriminated against by others in the community if they know about her/his problems? (Yes/No*).

### Scoring

The performance of each LLM was scored according to Jorm et al [43] and Caldwell and Jorm [38]. We then compared the performance of the LLMs to the norms of 324 mental health nurses, 228 clinical psychologists, 567 psychiatrists, 424 GPs, and 982 people from the general public, as collected in Australia [38,43]. Q5, which evaluated the positive and negative long-term outcomes, was calculated according to Caldwell and Jorm [38]. Each of the 10 statements was scored as follows: 1=more likely, 0=just as likely, and −1=less likely. The answers were then summed up, such that each positive and negative long-term outcome score ranged from −5 to 5.

### Statistical Analysis

The likely outcomes with and without professional treatment for the 2 vignettes, as evaluated by the LLMs, mental help professionals, and the general public (reported by Caldwell and Jorm [38,43]), were analyzed using 1-way ANOVA, with Fisher least significant difference applied as a post hoc analysis. The differences between the LLMs in positive and negative long-term outcomes were compared using 1-way ANOVA, with Fisher least significant difference applied as a post hoc analysis. Given the significant clinical implications of discrepancies between the evaluations of the LLM models and the professional assessments, we opted for a post hoc approach that minimizes the risk of type II errors or false negatives.

### Ethical Considerations

This study was exempt from ethical review since it only evaluates AI chatbots and no human participants were involved.

## Results

For all of the vignette cases, all 4 LLMs recognized schizophrenia as the primary diagnosis and suggested a combination of antipsychotic drugs and psychotherapy as the preferred treatment.

### Likely Outcome With Professional Treatment

**Figure 1.**
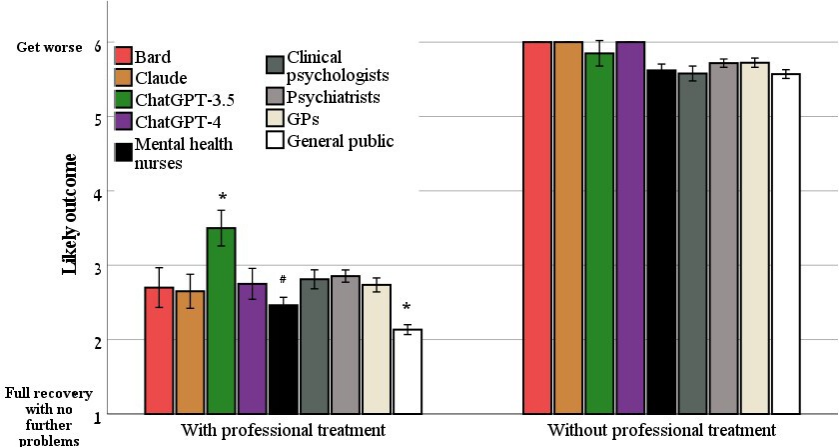
The likely outcome for schizophrenia, with and without professional treatment, as evaluated by large language models, mental health professionals, and the general public (mean and SE). *P<.05. GP: general practitioner.

Table 1 delineates the distribution of outcomes selected by LLMs, mental health professional groups, and the general public for a vignette describing an individual diagnosed with schizophrenia after receiving professional treatment. ANOVA analysis revealed significant differences in the selected outcomes across the 8 groups (*F*_8,2601_=33.66; *P*<.001). Post hoc analysis yielded the following insights. (1) The ChatGPT-3.5 model offered a distinctively pessimistic prognosis, significantly differing from the outcomes chosen by all the other LLMs (*P*=.02 to .007), the professional groups (*P*=.005 to <.001), and the general public (*P*<.001). (2) ChatGPT-4, Claude, and Bard projected more pessimistic prognosis outcomes than the general public (*P*=.02 to .007), whereas their projections were congruent with those from all the professional groups (all *P*>.05). A direct comparison of the projections of ChatGPT-4, Claude, and Bard yielded no significant differences (all *P*>.05; Figure 1 and Table 2).

**Table 1.** The likely outcome for schizophrenia, with and without professional treatment, as evaluated by LLMs^a^, mental health professionals, and the general public.

Professional treatment and outcome		ChatGPT-3.5 (n=20), n (%)	ChatGPT-4 (n=20), n (%)	Bard (n=20), n (%)	Claude, (n=20) n (%)	General public (n=982), %^b^	Nurses (n=324), %^b^	Clinical psychologists (n=228), %^b^	Psychiatrists (n=567), %^b^	GPs^c^ (n=424), %^b^
**With professional treatment**										
	Full recovery with no further problems	0 (0)	0 (0)	1 (5)	0 (0)	29.8	8.8	3.1	2	3.1
	Full recovery, but problems would probably reoccur	0 (0)	5 (25)	4 (20)	(35)	44.4	61.4	49.1	51.6	56.1
	Partial recovery	10 (50)	15 (75)	15 (75)	(65)	10.2	4.1	11.9	5.7	5
	Partial recovery, but problems would probably reoccur	10 (50)	0 (0)	0 (0)	0 (0)	14.3	25.7	35.4	40.6	35.8
	No improvement	0 (0)	0 (0)	0 (0)	0 (0)	0.7	0	0.4	0.2	0
	Get worse	0 (0)	0 (0)	0 (0)	0 (0)	0.6	0	0	0	0
**Without professional treatment**										
	Full recovery with no further problems	0 (0)	0 (0)	0 (0)	0 (0)	1.1	0	0	0	1.1
	Full recovery, but problems would probably reoccur	0 (0)	0 (0)	0 (0)	0 (0)	1.7	0.9	0.9	0.7	1.7
	Partial recovery	0 (0)	0 (0)	0 (0)	0 (0)	1.8	0.6	0.9	0.9	1.8
	Partial recovery, but problems would probably reoccur	0 (0)	0 (0)	0 (0)	0 (0)	4.9	11.3	9.1	5.7	4.9
	No improvement	3 (15)	0 (0)	0 (0)	0 0)	15.1	9.5	17.8	11	15.1
	Get worse	17 (85)	20 (100)	20 (100)	20 (100)	75.4	77.7	71.3	81.8	75.4

a LLM: large language model.

b As reported by Caldwell and Jorm [38].

c GP: general practitioner.

**Table 2.** Least significant difference post hoc analyses for LLMs^a^, mental health professionals, and the general public in assessing the outcome of schizophrenia with and without treatment.

*P* values			ChatGPT-3.5	ChatGPT-4	Bard	Claude	General public	Nurses	Clinical psychologists	Psychiatrists	GPs^b^
**With professional treatment**											
	ChatGPT-3.5		—^c^	.02	.01	.007	<.001	<.001	.003	.005	<.001
	ChatGPT-4		.02	—	.87	.75	.007	.21	.79	.64	.95
	Bard		.01	.87	—	.87	.01	.30	.63	.49	.87
	Claude		.007	.75	.87	—	.02	.41	.49	.37	.70
	General public		<.001	.007	.01	.02	—	<.001	<.001	<.001	<.001
	Nurses		<.001	.21	.30	.41	<.001	—	<.001	<.001	<.001
	Clinical psychologists		.003	.79	.63	.49	<.001	<.001	—	.58	.08
	Psychiatrists		.005	.64	.49	.37	<.001	<.001	.58	—	.06
	GPs		<.001	.95	.87	.70	<.001	<.001	.08	.06	—
**Without professional treatment**											
	ChatGPT-3.5		—	.55	.55	.55	.12	.21	.14	.46	.48
	ChatGPT-4		.55	—	>.99	>.99	.02	.04	.02	.11	.13
	Bard		.55	>.99	—	>.99	.02	.04	.02	.11	.13
	Claude		.55	>.99	>.99	—	.02	.04	.02	.11	.13
	General public		.12	.02	.02	.02	—	.35	.89	<.001	<.001
	Nurses		.21	.04	.04	.04	.35	—	<.001	.07	.07
	Clinical psychologists		.14	.02	.02	.02	.89	<.001	—	.03	.03
	Psychiatrists		.46	.11	.11	.11	<.001	.07	.03	—	<.001
	GPs		.48	.13	.13	.13	<.001	.07	.03	<.001	—

a LLM: large language model.

b GP: general practitioner.

c Not applicable.

### Likely Outcome Without Professional Treatment

Table 1 also delineates the distribution of outcomes selected by LLMs, mental health professional groups, and the general public for a vignette describing an individual with schizophrenia who did not receive professional treatment. All groups indicated that without treatment, the person with schizophrenia would show no improvement or would get worse. ANOVA analysis revealed a significant difference in the selected outcomes across the 8 groups (*F*_8,2601_=4.07; *P*<.001). Post hoc analysis yielded the following insights. (1) The ChatGPT-4, Claude, and Bard models offered a distinctively pessimistic prognosis, significantly differing from the outcomes chosen by mental health nurses (*P*=.04), clinical psychologists (*P*=.02), and the general public (*P*=.11) but not significantly different from the outcomes selected by ChatGPT-3.5, psychiatrists, and GPs (all *P*>.05). Direct comparison between ChatGPT-4, Claude, and Bard yielded no significant differences in prognosis (all *P*>.05). (2) No significant difference was observed between ChatGPT-3.5, the professional groups, and the general public (all *P*>.05; Figure 1 and Table 2).

### Long-Term Outcomes

**Figure 2.**
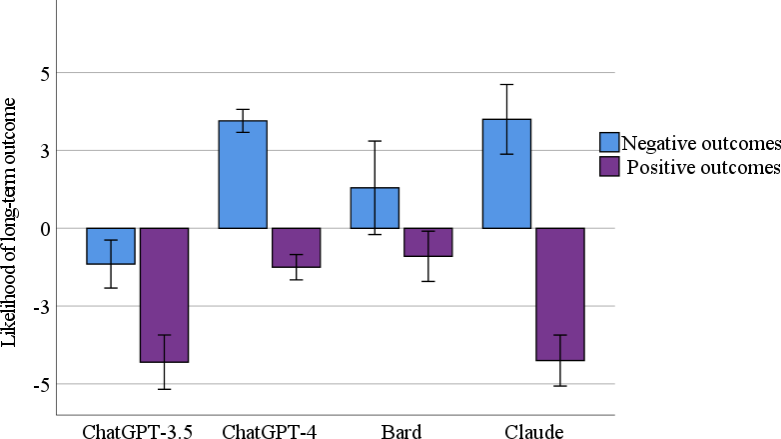
The positive and negative long-term outcomes evaluated by large language models (ChatGPT-3.5, ChatGPT-4, Bard, and Claude; mean and SE).

Figure 2 illustrates the LLMs’ output concerning positive and negative long-term outcomes. ANOVA analysis revealed a significant difference in the negative outcomes selected across the 4 LLMs groups (*F*_3,76_=18.32; *P*<.001). ChatGPT-4 and Claude indicated a significantly higher likelihood of negative long-term outcomes for patients after professional treatment than Bard and ChatGPT-3.5 (ChatGPT-4 vs Bard: *P*=.004; ChatGPT-4 vs ChatGPT-3.5: *P*<.001; Claude vs Bard: *P*=.003; Claude vs ChatGPT-3.5: *P*<.001). In addition, Bard was significantly more pessimistic and indicated a higher likelihood of negative long-term outcomes than ChatGPT-3.5 (*P*=.001). ANOVA analysis revealed a significant difference in the positive outcomes selected by the 4 LLMs groups (*F*_3,76_=24.45; *P*<.001). ChatGPT-3.5 and Claude were significantly more pessimistic and indicated a lower likelihood of positive long-term outcomes for patients after treatment than Bard and ChatGPT-4 (ChatGPT-3.5 vs Bard: *P*<.001; ChatGPT-3.5 vs ChatGPT-4: *P*<.001; Claude vs Bard: *P*<.001; Claude vs ChatGPT-4: *P*<.001). No significant differences were found between ChatGPT-3.5 and Claude (*P*=.92) or between ChatGPT-4 and Bard (*P*=.51).

### Discrimination

For all the cases in the vignettes, all 4 LLMs determined that the person with schizophrenia described in the vignette would be discriminated against.

## Discussion

### Principal Findings

This investigation pursued 2 primary objectives. First, we aimed to evaluate how various LLMs assessed the prognosis of individuals with schizophrenia compared to the evaluations of mental health professionals (mental health nurses, clinical psychologists, psychiatrists, and GPs) and the views of the general public. Second, we sought to compare these assessments of prognosis as well as positive and negative long-term outcomes across the different types of LLMs.

The academic discourse in contemporary schizophrenia research often focuses on the deployment of AI within professional scientific contexts, yet it seldom addresses the accessibility of AI to the general public or the patient population. LLMs are being used today by hundreds of millions of users worldwide, including patients and clinicians. In the mental health field, this widespread use has awakened an urgent need to examine the quality of clinical information these systems provide on various medical issues, such as treatment strategy recommendations [24], risk assessment [23,25], and the interpretation of emotional states [20,21]. Machine learning algorithms possess the capability to discern nuanced variables associated with divergent disease trajectories [44]. Such algorithms facilitate probabilistic prediction of specific outcomes at the individual level, as well as the identification of distinct subgroups within a broader diagnostic category [45]. Consequently, machine learning methodologies hold promise for aiding clinicians in formulating individualized interventions, thereby mitigating the risk of a detrimental disease progression [46]. This study examines an issue not previously investigated—the ability to predict the clinical prognosis of a severe chronic illness such as schizophrenia using LLMs.

### Likely Outcome With Professional Treatment

In this study, we identified significant differences in the outcomes suggested across the 8 groups. The ChatGPT-3.5 model exhibited a notably pessimistic prognosis for individuals with schizophrenia with professional treatment relative to all other LLMs, professional groups, and the general public. Given the widespread use of ChatGPT-3.5, these findings have substantial clinical implications. Any inclination toward pessimistic forecasting might influence a patient’s willingness to undergo treatments, including both antipsychotic medication and psychotherapy, particularly in the context of schizophrenia. If patients or their families consult the ChatGPT-3.5 model for prognostic insights, these less-than-optimistic outcomes might sway their decision-making regarding whether to initiate or continue therapeutic interventions. The literature previously highlighted challenges in adherence to antipsychotic and psychotherapy treatments due to their cost and other factors [47,48]. Additionally, more negative perceptions of potential treatment outcomes might inadvertently influence the effectiveness of the therapeutic process, regardless of the mode of intervention.

The congruence between the prognostic assessments of various LLMs models (ChatGPT-4, Bard, and Claude) and those of clinical professionals is encouraging. From a clinical perspective, precise prognostication is paramount. It not only informs clinicians in tailoring interventions that balance potential risks and benefits but also empowers patients with the knowledge needed to make informed choices about their treatments while considering the inherent risks associated with the intervention and the disease’s progression. The finding that 3 prominent LLMs yielded comparable estimates that align closely with the evaluations of 3 groups of experienced professionals (GPs, psychiatrists, and clinical psychologists) offers a foundation for optimism. Such consistency in predictive capabilities suggests the potential for integrating these insights into clinical decision support systems, reinforcing the centrality of accurate prognostication in medical decision-making.

This observation substantiates initial results in the domain of mental health research gleaned from the use of the ChatGPT-3.5 model. Existing methodologies often exhibit constrained predictive proficiencies. In a recent study, Elyoseph and Levkovich [25] found that ChatGPT-3.5 often underestimated the risk of suicidal ideation, thus calling into question its reliability in such critical assessments. Another study by Imran et al [49] posited that while ChatGPT may significantly influence pediatric and adolescent mental health care as a supplementary tool, it would be inadvisable and impracticable to contend that it could entirely supplant human clinical discernment. Indeed, although the utility of ChatGPT in mental health spheres appears promising, significant reservations remain. Another study suggested that ChatGPT-4 estimates the likelihood of suicide attempts in a manner akin to evaluations provided by professionals, whereas ChatGPT-3.5 frequently underestimates suicide risk [23]. For instance, ChatGPT’s learning mechanisms, which rely on web-based data and human feedback, have the potential to disseminate inaccurate or inappropriate guidance if not rigorously evaluated. Such drawbacks are especially disturbing when considering their impact on individuals grappling with mental health disorders [50].

ChatGPT-4, Bard, and Claude have each instituted measures aimed at forestalling malevolent use and attenuating biases inherent in their respective models; however, challenges persist in ascertaining how these technologies should be responsibly used. The intrinsic worth of the generative output produced by LLMs is the subject of scholarly contention. Some researchers, such as Winkler et al [51], posit that LLMs may actually constitute a deceptive or even perilous risk due to their capacity to fabricate an appearance of comprehension, sentience, and analytical depth in the absence of an authentic world model. Medical studies that compared different LLMs found that ChatGPT-4 and Bard aligned with doctors’ diagnoses [52]. Another study [53] sought to assess the performance of 4 LLMs (Claude, Bard, ChatGPT-4, and New Bing) in the context of medical consultations related to urolithiasis. Simulated clinical scenarios revealed that all the models except Bard provided relatively competent answers. Claude consistently excelled in various evaluative metrics, whereas ChatGPT-4 ranked second in accuracy and demonstrated stable output across tests.

### Likely Outcome Without Professional Treatment

In this study, all groups expressed the belief that in the absence of medical intervention, an individual diagnosed with schizophrenia would either demonstrate no improvement or would deteriorate. This assessment is similar to the evaluation of psychiatrists and GPs and is consistent with the literature and clinical knowledge [38,43]. We suggest that these assessments, although slightly more pessimistic than those of clinical psychologists, nurses, and the general public, have a positive influence because they emphasize the risk of untreated illness and indirectly encourage treatment.

To the best of our knowledge, no studies have examined comparison between these LLMs in this context of mental health. Nevertheless, initial studies that compared professionals in the field of therapy and medicine reinforce these findings. For example, in a scholarly investigation encompassing 82 clinical descriptions [54], the diagnostic accuracy rates of physicians were found to surpass those of Bard. This outcome indicates that Bard needs further enhancement and fine-tuning in its diagnostic proficiencies. Another possible explanation for the findings is that there are fundamental differences between the various algorithms. These algorithms were trained on different amounts and qualities of data, underwent different processes of elimination, and use distinct strategies for receiving feedback from system users [55].

### Long-Term Outcomes

In the case of assessing long-term outcomes, 3 of the models—ChatGPT-4, Bard, and Claude—paralleled the conclusions reached by mental health professionals [38,43]. The models pointed to a higher likelihood of negative long-term outcomes and a decreased probability of positive ones. ChatGPT-3.5, which projected a decline in negative long-term symptoms over time, is an anomaly. Apart from this exception, the evaluations of the 3 models and the determinations of mental health specialists exhibit consistent alignment. An analysis of the differences among the 3 revealed that Claude has the most conservative or pessimistic stance, ChatGPT-4’s predictions are midway between pessimistic and optimistic, and Bard exhibits the most optimistic forecasting. These results again underscore the potential of LLMs models to offer prognostic insights that might be incorporated into future medical decision-making processes.

### Real-World Application Potential

This investigation presents initial discoveries regarding the potential of LLMs in offering prognostic forecasts for schizophrenia. It is of utmost importance to approach these findings with caution, considering the potential fragility of these models over time and the limited scenarios analyzed in the study, which do not fully encompass the range of symptoms, medical histories, and individual variations. Moreover, the study does not explore LLM predictions across various treatment strategies. Nevertheless, by adopting a careful approach, we strive to elucidate the future potential of using these capabilities in real-world clinical settings through further research. One potential avenue for integrating LLMs into clinical practice is by using them as a “co-pilot” that aids clinicians by providing pertinent information. For instance, LLM systems could potentially offer prognostic evaluations based on symptom descriptions during intake, summarized reports of visits, or transcriptions of conversations with clinicians. Clinicians could use this information to align expectations with patients regarding their prognosis or to tailor treatment, taking into account the implications on patients’ lives. It is important to note that although theoretically possible, the ability of AI to provide patient-specific prognoses, which could potentially enhance treatment protocols and align expectations between patients and caregivers, remains to be empirically demonstrated. Another option is the direct use of LLMs by patients and family members as part of a psychoeducational process to familiarize themselves with the illness and its potential consequences. This approach can enhance collaboration and engagement in the treatment process.

Lastly, AI systems have the capability to process auditory information, such as a case narrative, and generate a prognosis based on it. There exists potential to convert this qualitative, subjective information into an objective, mathematical analysis. Essentially, AI takes the primary input received by a physician—the patient’s narrative of their illness—and objectively analyzes it rather than subjectively. This has the potential to enhance the reliability of assessment processes in the field of psychiatry. By combining such tools with additional data, it is possible that prognoses can be further improved. Future research can explore the combined impact of artificial and human predictions and incorporate questionnaires to refine the predictive outcome of disease progression.

### Limitations

This research is not without limitations that necessitate explicit acknowledgment. First, since the study tested the performance of LLMs at one point in time, it is necessary to examine the consistency of the results when software updates are released. Second, the data pertaining to AI were juxtaposed with information gleaned from a sample of professionals and the general populace in a single study in Australia. This sample, however, does not offer global representation. Future investigations are recommended to encompass a more extensive array of variables, such as socioeconomic indicators, cultural determinants, and mental health histories, particularly with regard to recovery from schizophrenia. Furthermore, the vignettes used in the study, including those featuring individuals with schizophrenia, fail to present a nuanced, ongoing, and comprehensive medical treatment context. They also do not include variables that would be readily available to medical professionals during therapeutic sessions. To enhance the generalizability and rigor of subsequent studies, it is advised to incorporate additional variables, deploy more sophisticated language models, evaluate data at varying temporal intervals, and juxtapose the findings with a more diverse assortment of clinical samples. An additional constraint involves ethical considerations in professionals’ use of AI. The literature reveals public skepticism and concerns about medical inaccuracies and potential discrimination [56,57]. Ethical issues such as patient autonomy and health disparities necessitate exercising caution in AI’s medical applications [58-62]. Lastly, the rapidly evolving landscape of AI poses an inherent obstacle to drawing conclusions about the technology’s long-term, stable capabilities. To address this concerns, future research is required. To enhance the accuracy of LLMs in psychiatric assessment, future research should focus on enriching training data sets with specialized, targeted data, including historical clinical knowledge and detailed patient histories. Validating these models against current clinical practices and decisions made by practicing psychiatrists can provide a practical benchmark for their performance. Additionally, exploring technological advancements in AI, particularly in deep learning, can refine LLMs to process complex psychiatric data more effectively. Modifying prompts and inputs to better reflect psychiatric assessments can also improve the models’ understanding and interpretation of clinical scenarios. Interdisciplinary collaboration involving AI researchers, clinicians, and ethicists is essential to align the development of LLMs with clinical needs and ethical standards. Investigating the integration of LLMs with human expertise, through interactive systems that allow clinicians to provide feedback on LLM predictions, is crucial for a dynamic learning process. Exploring the use of LLMs across diverse clinical environments and patient populations can help identify and mitigate potential biases, ensuring equitable and broadly applicable models. Longitudinal studies tracking LLM performance over time in various clinical contexts will provide insights into long-term efficacy and areas for improvement. These research initiatives can significantly advance the field of LLMs in psychiatry, enhancing their accuracy, reliability, and practical utility in clinical settings.

### Conclusion

This study offers novel and clinically relevant insights into the assessment capabilities of prominent LLMs regarding the prognosis and long-term outcomes of schizophrenia. The findings highlight both the promise and current limitations of AI in augmenting clinical evaluations. Further research is warranted to refine the algorithms and better integrate human expertise, thereby maximizing the judicious and ethical use of AI in mental health care.

## Abbreviations

AI: artificial intelligence
GP: general practitioner
LLM: large language model
